# Scenarios in tropical forest degradation: carbon stock trajectories for REDD+

**DOI:** 10.1186/s13021-017-0074-0

**Published:** 2017-03-09

**Authors:** Rafael B. de Andrade, Jennifer K. Balch, Amoreena L. Parsons, Dolors Armenteras, Rosa Maria Roman-Cuesta, Janette Bulkan

**Affiliations:** 1 Geography Department, University of Colorado-Boulder, Boulder, USA; 2 Geography Department, Pennsylvania State University, State College, USA; 3 Departamento de Biologia, Universidad Nacional de Colombia, Bogotá, Colombia; 4 WU Environmental Sciences, Wageningen University and Research Centre, Wageningen, Netherlands; 5 Department of Forest Resources Management, University of British Columbia, Vancouver, Canada

## Abstract

**Background:**

Human-caused disturbance to tropical rainforests—such as logging and fire—causes substantial losses of carbon stocks. This is a critical issue to be addressed in the context of policy discussions to implement REDD+. This work reviews current scientific knowledge about the temporal dynamics of degradation-induced carbon emissions to describe common patterns of emissions from logging and fire across tropical forest regions. Using best available information, we: (i) develop short-term emissions factors (per area) for logging and fire degradation scenarios in tropical forests; and (ii) describe the temporal pattern of degradation emissions and recovery trajectory post logging and fire disturbance.

**Results:**

Average emissions from aboveground biomass were 19.9 MgC/ha for logging and 46.0 MgC/ha for fire disturbance, with an average period of study of 3.22 and 2.15 years post-disturbance, respectively. Longer-term studies of post-logging forest recovery suggest that biomass accumulates to pre-disturbance levels within a few decades. Very few studies exist on longer-term (>10 years) effects of fire disturbance in tropical rainforests, and recovery patterns over time are unknown.

**Conclusions:**

This review will aid in understanding whether degradation emissions are a substantial component of country-level emissions portfolios, or whether these emissions would be offset by forest recovery and regeneration.

## Background

Greenhouse gas emissions from tropical rainforest degradation are substantial. Of total emissions, degradation is responsible for at least one-fifth in the Brazilian Amazon [1], two-thirds in Indonesian forests [2], and almost half in African tropical forests [3]. In 2002, the International Tropical Timber Organization estimated that up to 8.5 million km^2^ of tropical forest and forest lands could be degraded [4]. In 2007, the Thirteenth Conference of the Parties (COP 13) to the United Nations Framework Convention on Climate Change (UNFCCC) explicitly made addressing forest degradation part of the proposed mechanism for reducing emissions from deforestation and forest degradation (REDD+). More recently, the COP 21 agreement signed in 2015 in Paris, urges the 195 participant parties to implement and support the existing framework for reducing emissions from deforestation and forest degradation. However, there is no consensus on what level or threshold of forest biomass loss and the persistence of that carbon loss constitutes degradation. The Intergovernmental Panel on Climate Change (IPCC) defines forest degradation as ‘direct human induced long-term loss (persisting for X years or more) of at least Y% of forest carbon stocks (and forest values) since time (T) and not qualifying as deforestation’ [5]. Recent monitoring plans adopted degradation as the ‘reduction of carbon stocks by at least 10% and persisting for 5 years or more’ [6]. Here, we review all studies that document any amount of decline in aboveground forest carbon stocks via logging or fire in tropical forests.

According to Herold et al. [7], it is estimated that disturbances result in annual degradation of approximately 1 million km^2^ of forests globally, an area 10 times greater than the one impacted by deforestation, and a total area up to 850 × 105 km^2^ [4]. Wildfires in intact Amazon forests affected ~80,000 km^2^ from 1999 to 2010 [8] which was greater than the area affected by deforestation [9]. Areas degraded by logging in tropical regions also can exceed the area deforested [10]. However, it is very uncertain how much carbon is actually emitted in the short- and long-term from degradation activities [11]. Additionally, it is estimated that gross emissions from timber production are significant, but highly variable. For example, in 2005, the proportion of logging emissions relative to deforestation ranged from 6% in Brazil and Indonesia to about 30% in the Republic of Congo and Suriname to 68% in Malaysia [12].

However, the difficulty in defining and monitoring forest degradation has hindered policy efforts that attempt to limit degradation activities [13, 14]. Generally tropical forest degradation is defined as the substantial decline in forest structure or function over time due to human activities, without complete conversion to another land use, incorporating elements identified by the Intergovernmental Panel on Climate Change [5]. Activities that induce forest degradation include fire, logging, fuelwood extraction, and sub-canopy grazing and cultivation [15]. Forest degradation can be slow or rapid, distributed or concentrated, but must result in net carbon emissions that are not recovered over short time scales through forest recovery and regeneration. Based on ecological theory, our expectations are that single or light disturbances may allow for quick recovery of forest structure, whereas intense, repeated, or synergistic disturbances may lead to substantial forest decline and potentially lower carbon states. Currently, forest degradation is most easily measured by change in canopy cover, biomass, or annual productivity (compared with the same measures in natural, mature forest stands of the same ecoregion). Forest functions, such as carbon, water, or nutrient cycling, however are more difficult to measure. Given the availability of more information on changes in canopy cover and biomass for tropical forests, and type of disturbance, we focus this review on best available science that documents changes in forest structure due to logging and fire degradation.

Biomass depletion from logging activities comprise much more than the mere quantity of extracted timber. Most of the carbon losses come from felled trees that are abandoned in the forest, and trees incidentally damaged during felling. Aboveground and belowground biomass of the stump and crown left as deadwood, plus dead and damaged surrounding trees, can make up to 51% of the total carbon emissions in a harvesting activity [12]. This is followed by the carbon emissions associated with logging infrastructure, i.e. roads, skid tracks, and logging decks necessary for the harvesting. Infrastructure can make up to 45% of total logging emissions in countries such as Indonesia [12]. Much less information on carbon stock depletion is available for fire-affected tropical forests. Intense slash-and-burn fires can convert almost 40% of the initial above-ground carbon to carbon emissions, and only 2% to permanently sequestered charcoal [16]. Small surface fires, more associated with forest degradation, usually burn only the leaf litter as an immediate result [17], but subsequent tree mortality can deplete standing biomass up to many years post-fire [17–19].

There has been substantial work on tropical rainforest disturbance and recovery [20], yet this information could be better synthesized to provide practical guidelines for specifically understanding degradation in the context of REDD+. Critical information to synthesize across tropical regions is what is known about initial gross and longer-term net degradation emissions, the spatial distribution of degradation, and what degradation scenarios lead to substantial and semi-permanent carbon losses.

As an example, there has been considerable attention and resources devoted to understanding whether the Amazon forest biome is a carbon sink or source [21]. The net effect of Amazon deforestation and reforestation results in an annual net C source of 0.15–0.35 Pg C, but adding fire and logging extends that range to an annual net release of 0.2–0.8 Pg C [22]. All forms of degradation have lower emissions per area than deforestation, but can be more extensive—thereby surpassing total emissions from deforestation in certain years. For example, during the 1997–98 El Nino drought event, 39,000 km^2^ of otherwise intact Amazon forests burned—twice the area annually deforested in Brazil (1988–2004—releasing 0.05–0.33 Pg of carbon [23]. Between 1999 and 2002, more than 12,000 km^2^ per year were selectively logged in the Amazon, releasing a gross flux of approximately 0.1 billion metric tons of carbon [1].

In Brazil, recent (2009–2012) deforestation rates were 80% less than the average for 1988–2008 [9], which may mean that degradation emissions will be a more substantial component of this country’s future emissions portfolio. At this juncture, estimates of the recovery rates and trajectories of emissions after degradation are a critical piece of information for REDD+ negotiations in many tropical countries. A mass of evidence points to repeated disturbances, including re-entry logging in Borneo, substantially reducing forest carbon stocks, limiting forest recovery, or leading to an alternate lower-carbon vegetation type. Field-based studies in the southern Amazon show that repeated fire disturbances can lead to at least a 20% increase in tree mortality, compared with a single fire event [19]. Chronosequence studies of abandoned pastures in the Amazon show that the rates of secondary forest recovery are negatively correlated with the number of previous burns [24]. Further, forest edges exposed to repeated burns are quickly dominated by non-native grasses associated with pastures [25, 26], leading to a much lower carbon state.

Our objective is to review the current available scientific information on aboveground carbon stock trajectories through time after logging and fire degradation in tropical forests. We focus on the dynamics of biomass loss and recovery after a specific type of degradation occurs. That way, we hope to inform REDD+ policy discussions and country-level decisions on whether to invest on monitoring and prevention of forest degradation.

## Methods

The IPCC (2003) recommends two different approaches to calculating emissions: the stock-difference method and the gain-loss method. The former builds on traditional forest inventories to estimate sequestration or emissions, while the latter builds on an understanding of forest dynamics, incorporating carbon accumulation through forest recovery and regeneration. The stock-difference method measures the actual stock of biomass at the beginning and end of the accounting period. The gain-loss method estimates biomass as the net balance of additions to and removals from a carbon pool, i.e. the balance between growth and loss from harvesting, decomposition, or burning [27]. The latter is much more difficult to quantify given that it is dependent on estimating tree increment and ingrowth of new stems. Here, we report on studies that mostly provide stock-difference estimates.

### Literature survey and calculation descriptions

**Fig. 1:**
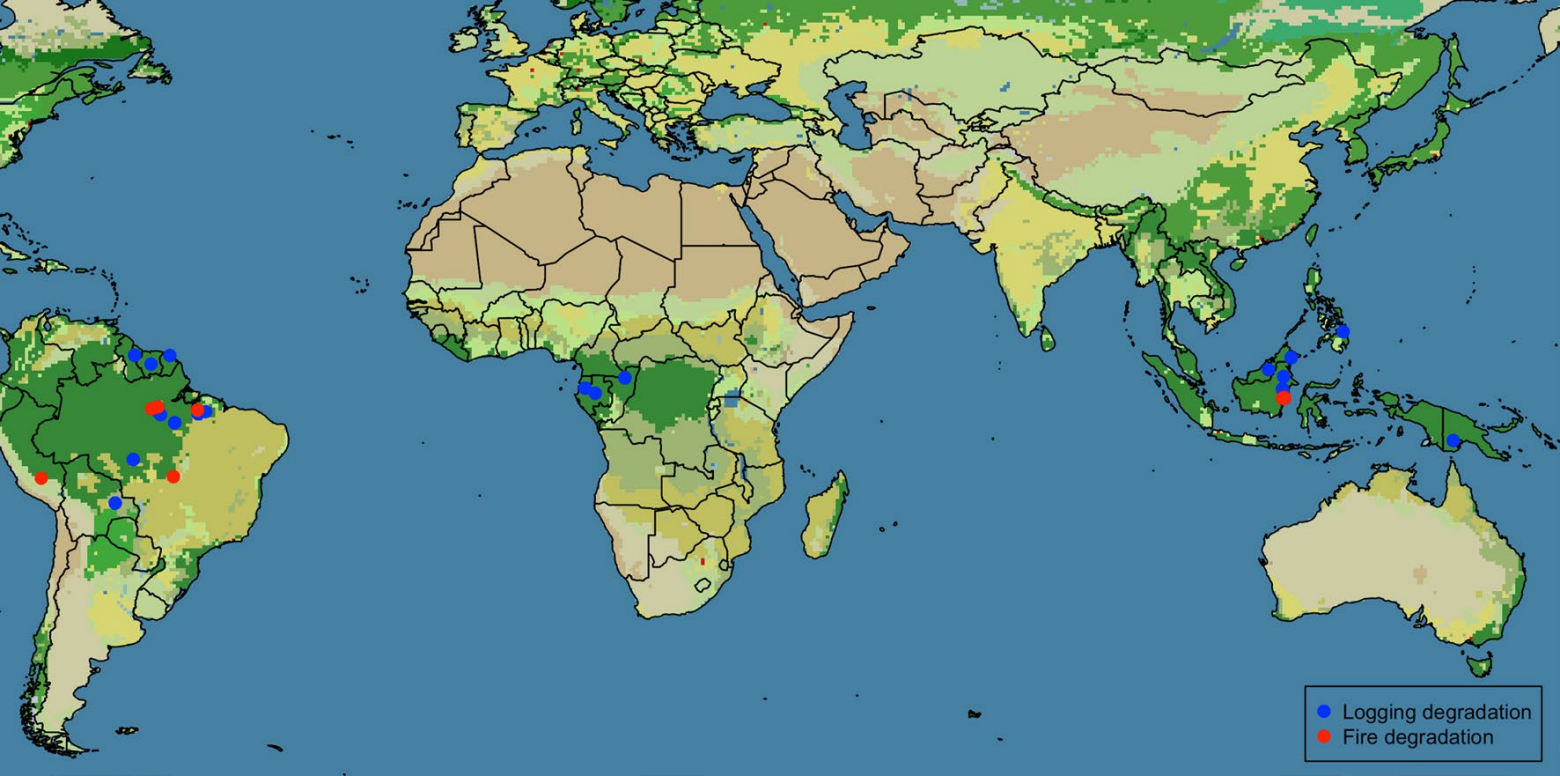
Field-based studies on carbon emission or reduction from logging (*blue dots*) and fire degradation (*red dots*) in closed canopy tropical forest (*dark green*)

For our meta-analyses, we identified case studies and reviews on depletion of carbon stocks in tropical rainforests due to logging activities and fire disturbance. For a consistent analysis on aboveground carbon stock trajectories, all standing carbon or emission data used in our review necessarily contains an estimation of time since disturbance, and comparison with pre-disturbance or control forest. Using the search terms “redd”, “degradation”, “fire”, and “logging” in search engines Web of Science and Google Scholar, and following cited references within the studies, we compiled data from 54 published papers that matched our requirements. We limited our search to surveys on closed canopy forest, but did not distinguish among subtypes of forest (e.g. montane, peatland, transitional). We used data from both remote sensing or in situ field-based measurements on aboveground biomass. Many studies could not be included in our analyses for not having a clear ‘time since disturbance’ estimate [28, 29], a well-defined type of degradation [30–32], or baseline values [8, 33]. Additionally, remote-sensing studies covering areas in a regional or continental scale were not included [34]. Out of the 54 studies used in our review, 24 were field-based and mostly concentrated on South America and Southeast Asia (Fig. 1). As several studies report carbon and biomass in different ways, we standardized the carbon emission data by converting it to MgC/ha when necessary. For most studies, carbon emission was calculated as the difference between the standing biomass or carbon (assumed to be half of total biomass) in the degraded area and in an intact control forest. We also calculated the percentage of emitted carbon relative to intact standing carbon in aboveground biomass. If reported, we calculated minimum, maximum and average carbon emissions in MgC/ha. Whenever available, we recorded time between the degradation and carbon/biomass assessment, forest type, and total area encompassed by the study. For studies using stem diameter as a proxy for standing biomass, we used only the data of stems DBH > 10 cm whenever reported separately. Trajectories of percent aboveground carbon along years since degradation were plotted using default *loess* smoothing, with visual selection of *span* value, in package *ggplot2* for R [35].

## Results

**Fig. 2:**
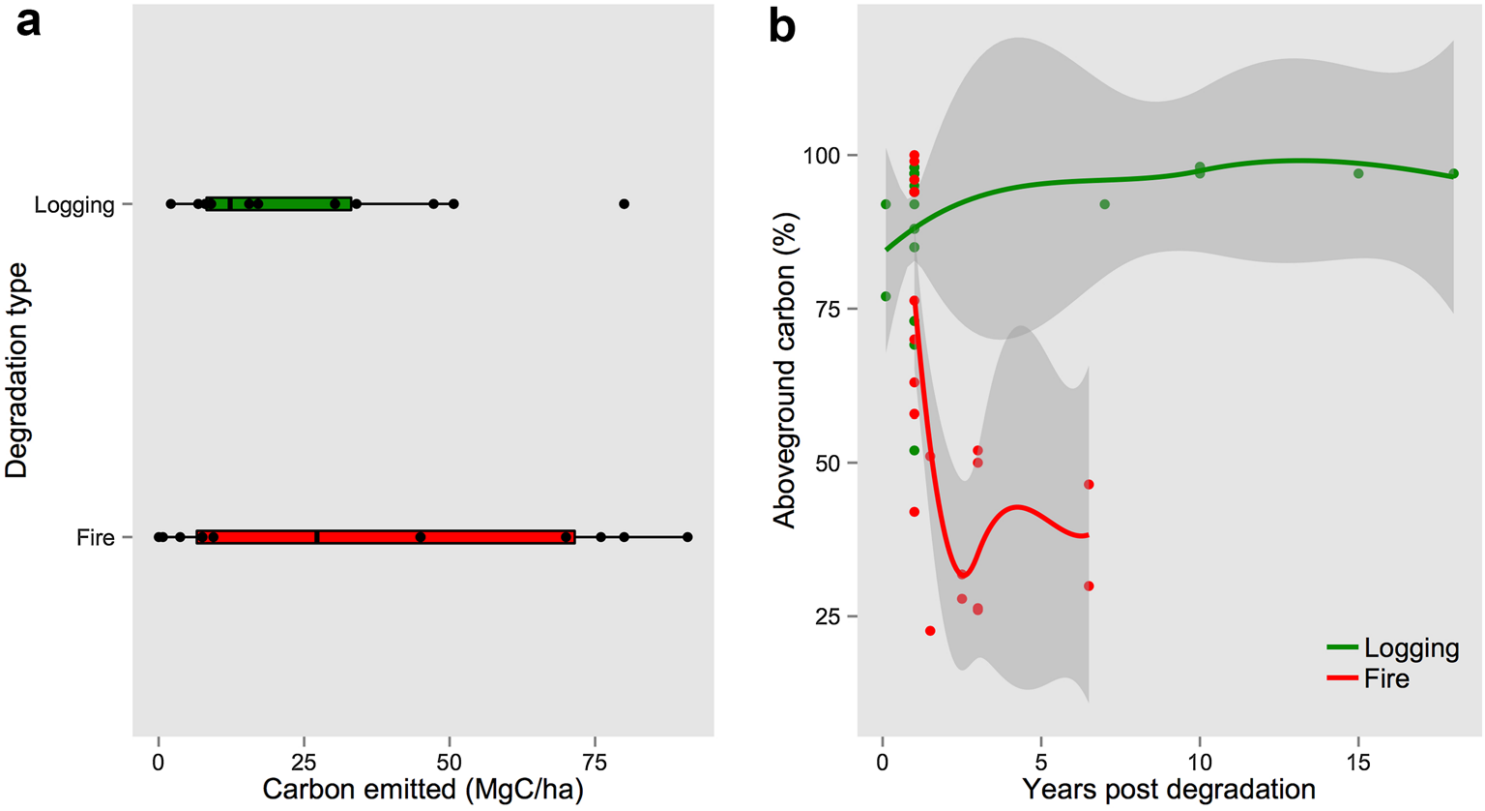
**a** Short-term (1 year or less) carbon emissions after logging (average = 23.5 MgC/ha, minimum = 2.1 MgC/ha, maximum = 80.0 MgC/ha), and fire disturbance (average = 36.3 MgC/ha, minimum = 0.03 MgC/ha, maximum = 90.9 MgC/ha). **b** Aboveground carbon stock (in % of original/undisturbed forest) trajectories after logging (*green*) and fire (*red*), collected from 31 studies reporting carbon or biomass in degraded forests. *Shaded areas* represent 95% confidence intervals of loess smoothing

Of all studies on aboveground carbon emissions from logging, average emissions was 19.9 MgC/ha (SD = 30.0), with maximum emission reported was approximately 80 MgC/ha [36]. Average emissions reported in all fire disturbance studies was 46.0 MgC/ha (SD = 29.9), with a maximum of 125.9 MgC/ha [37]. Twelve studies (total of 28 data points) on logging and eight studies (total of 20 data points) on fire degradation in tropical forests reported precise time since degradation and carbon stocks/emissions. Most of them, however, assessed aboveground carbon stocks/emissions within the first year since the degradation occurred (Fig. 2a). Average time since disturbance for data was 3.22 for logging and 2.15 years for fire. Logging had a lower overall short-term (<1 year) emission (average = 23.5 MgC/ha, minimum = 2.1 MgC/ha, maximum = 80.0 MgC/ha), while fire disturbance had higher emissions and variability (average = 36.3 MgC/ha, minimum = 0.03 MgC/ha, maximum = 90.9 MgC/ha).

Loess smoothing curves for reports of aboveground carbon stocks along years post-degradation (Fig. 2b) showed single or one-time logging (28 observations, span = 0.89, residual standard error = 11.44) reducing initial aboveground carbon stocks to approximately 80%, with values close to recovery after one decade. Fire (20 observations, span = 1.1, residual standard error = 18.37), however, depletes initial aboveground carbon to below 50%, and showed no sign of recovery within the 5 years post-fire documented in the reviewed studies. Relative to initial aboveground carbon, studies reported a minimum of 54% [38] and maximum of 98.1% [39] remaining carbon after logging, and a minimum of 22% [40] and maximum of 100% [16] after fire disturbance.

## Discussion

Our meta-analysis shows that degradation-related emissions of aboveground carbon from in tropical forests from logging range from 2.1 MgC/ha [41] to 116 MgC/ha [42]—and from fire ranged from 0.3 MgC/ha [16] to 125 MgC/ha [37]. Since we limited our meta-analysis to standing aboveground carbon, these numbers are likely and underestimation when compared with studies that incorporate other carbon pools, such as coarse woody debris [43]. Sasaki et al. [44] show reduced-impact logging activities reducing stocks in more than 50 MgC/ha on average. The pattern of emissions from logging and fire are quite different and relate to the type of disturbance, the process by which live trees are killed, and whether ecosystems are vulnerable. Logging emissions are concentrated at the beginning of the disturbance event, as biomass is removed and there is collateral damage to nearby trees. Fire emissions tend to peak several years after the disturbance event [18, 19], as it tends to take trees a long time to die from fire damage and consequent other insults such as disease, drought, and insect infestations [45]. Additionally, studies in tropical forests indicate a possible transition to lower carbon stable states dominated by grasses [26]. Although based on very limited data, the dip in the average aboveground carbon 3 years after disturbance in our estimated trajectory does agree with results showing late mortality of large thick-barked trees [18, 19].

The intensity of the disturbance event relates strongly to the amount of carbon lost and the recovery time. Within logged and unlogged forests, standing carbon, forest structure, and biodiversity can be highly variable, ultimately affecting ecological functioning and recovery time [46, 47]. A more detailed study on reduced impact logging and recovery periods by [48] (not included in our meta-analysis) shows the intensity of the logging activity (i.e. the volume extracted) as the main predictor of recovery time in tropical forests. The study shows that a 10% loss of original biomass due to one-time, not repeated, logging takes an estimated 10 years to recover, while a loss of 50% can take up to 75 years. Most studies in our analyses have an initial carbon loss after logging below 25%, and the trajectory suggests carbon stock recovery within 10–20 years (Fig. 2b). The intensity of fire events, on the other hand, can be much more variable. Seasonality, air humidity, temperature, winds, and previous disturbances can significantly shape the severity, area, and duration of a forest fire [17, 49]. Despite the lack of long-term data on fire-degraded forests, our analysis indicates a highly unlikely scenario of carbon stock recovery after burns. Therefore, in any degradation monitoring scheme, monitoring the intensity of the event is a critical piece of information for determining long-term carbon stocks and rates of change.

It is difficult to assess with current evidence how logging and fire degradation in tropical forests may lead to a more permanent, lower carbon state—either through arrested succession, a switch to an alternate vegetation state, or facilitation of future deforestation. The studies reviewed here suggest that type and intensity are the determinant factors of recovery pattern and, consequently, possible conversions to lower-carbon states. One study from the southeastern Amazon shows that combined disturbance from drought and fire can lead to a lower carbon state, i.e. reduction of 90% of aboveground biomass, due to a transition from forest to a novel grassland state [50].

Our compilations highlight the limitations of available data, especially for fire disturbance, but hopeful progress is being made. Recent projects producing “wall-to-wall” biomass maps (e.g. Global Carbon Project by the Woods Hole Research Center) can benefit long-term monitoring of post-fire responses. Notably, the geographic distribution of the handful of studies that lent themselves to this review span a great area, but leave many gaps in our understanding across the many tropical forests types (Fig. 1). In particular, studies on African tropical forests are few and far between. On the other hand, this review focused on logging and fire, which are the main drivers of degradation in South American countries, whereas fuelwood extraction and charcoal production are the main drivers in African countries [51].

### Implications of our findings

The 2015 COP 21 agreement, recently signed by 195 countries, recognizes the importance of “(…) policy approaches and positive incentives for activities relating to reducing emissions from deforestation and forest degradation (…)” [52]. Although available studies indicate fire disturbance as a much worse issue in terms of carbon loss and persistence, it seems to be often ignored in discussions on curbing degradation emissions [53]. Future monitoring efforts should consider the importance of capturing the peak of emissions (e.g. immediately in the case of logging and some years post-fire in the case of fire). Our review agrees with other studies showing forest recovery of low-intensity logging within 50 years [48], but this is unknown for fire-related degradation. Understanding recovery intervals for logged forests is directly beneficial for management policies, such as allowable volume cut and cutting cycle intervals.

Forest fires are becoming more intense and frequent in many tropical biomes [54, 55], and having more data on persistence and recovery of burned forests would help establish effective policies for monitoring and regulating agricultural burning activities. Other forms of degradation, such as fuelwood collection and charcoal production, are major drivers of carbon loss on the African continent, but very little data is available for emissions from these activities. Recent advances in remote sensing of degradation, along with improved in situ carbon measurements, show promising possibilities for forest monitoring in the African continent (e.g. REDDAF project).

Beyond a better understanding of the idiosyncrasies in each degradation type, more information on how initial intensity predicts persistence can inform REDD+ policies. Forestry activities or disturbances should be prioritized in reports and government initiatives if they result in permanent or persistent carbon stock reduction. Understanding degradation in tropical forests can be a conceptually complicated problem, especially when dealing with logging and fire disturbances. Although tree fall gaps are known to be important components of forest dynamics [56], the canopy gaps produced by reduced impact logging can be substantially different from natural ones [57]. Similarly, due to the scope of our review, the studies selected comprise habitats in which fires can be part of their natural cycle. Many tropical forests, however, are increasingly under the threat of changing fire regimes due to positive feedback in ignition sources from human occupation, longer and drier dry seasons, and the increase of greenhouse gas emissions [49, 58]. In the context of recent climate change negotiations, we hope that this review of current available scientific data highlights our collective knowledge and identifies important gaps in understanding of degradation-related emissions and potential recoveries.
